# Construction of co‐expression modules related to survival by WGCNA and identification of potential prognostic biomarkers in glioblastoma

**DOI:** 10.1111/jcmm.16264

**Published:** 2021-01-15

**Authors:** Jing Zhou, Hao Guo, Likun Liu, Shulan Hao, Zhi Guo, Fupeng Zhang, Yu Gao, Zhi Wang, Weiwei Zhang

**Affiliations:** ^1^ Department of Oncology Shanxi Province Academy of Traditional Chinese Medicine Shanxi Province Hospital of Traditional Chinese Medicine Taiyuan China; ^2^ Shanxi University of Chinese Medicine Taiyuan China; ^3^ Department of Anesthesiology Shanxi Provincial People's Hospital Taiyuan China

**Keywords:** cell cycle, glioblastoma, hub gene, the Cancer Genome Atlas (TCGA), Weighted Gene Co‐expression Network Analysis (WGCNA)

## Abstract

Glioblastoma (GBM) is a malignant brain tumour with poor prognosis. The potential pathogenesis and therapeutic target are still need to be explored. Herein, TCGA expression profile data and clinical information were downloaded, and the WGCNA was conducted. Hub genes which closely related to poor prognosis of GBM were obtained. Further, the relationship between the genes of interest and prognosis of GBM, and immune microenvironment were analysed. Patients from TCGA were divided into high‐ and low‐risk group. WGCNA was applied to the high‐ and low‐risk group and the black module with the lowest preservation was identified which could distinguish the prognosis level of these two groups. The top 10 hub genes which were closely related to poor prognosis of patients were obtained. GO analysis showed the biological process of these genes mainly enriched in: Cell cycle, Progesterone‐mediated oocyte maturation and Oocyte meiosis. CDCA5 and CDCA8 were screened out as the genes of interest. We found that their expression levels were closely related to overall survival. The difference analysis resulted from the TCGA database proved both CDCA5 and CDCA8 were highly expressed in GBM. After transfection of U87‐MG cells with small interfering RNA, it revealed that knockdown of the CDCA5 and CDCA8 could influence the biological behaviours of proliferation, clonogenicity and apoptosis of GBM cells. Then, single‐gene analysis was performed. CDCA5 and CDCA8 both had good correlations with genes that regulate cell cycle in the p53 signalling pathway. Moreover, it revealed that high amplification of CDCA5 was correlated with CD8^+^ T cells while CDCA8 with CD4^+^ T cells in GBM. These results might provide new molecular targets and intervention strategy for GBM.

## 1 INTRODUCTION

Glioblastoma (GBM) is the most common malignant tumour of the central nervous system (CNS) in adults.^1^, ^2^ It comprises 45.2% of CNS tumours and 54% of all gliomas.^3^ Corresponding therapeutics, for instance, maximum surgical resection, comprehensive radiotherapy and chemotherapy have been applied to clinical practice. Yet the advances in treatment have not concomitant with prominent amelioration in outcomes until recently. The 5‐year survival rate is still very low.^4^, ^5^, ^6^ Plenty of studies have been conducted to identify the underlying pathogenesis mechanisms, however, it's still not been illuminated.^7^, ^8^, ^9^ Particularly, there are few related studies on the expression modules of GBM, which has brought certain difficulties to the identification of key genes in the occurrence and recurrence of disease. The potential heterogeneities and complexities of GBM make it difficult to identify reliable factors for determining effective clinical treatment. Hence, it is urgently needed to uncover efficient molecular targets which can clinically significance contribute to the personalized treatment and improve prognosis for GBM patients.

As a newly invented systematic biology approach, Weighted Gene Co‐expression Network Analysis (WGCNA), has been used to describe the connectivity of gene clusters inside the comprehensive network and assess the correlations of gene modules with different clinical features.^10^, ^11^ Distinguished from other analysis method, WGCNA hierarchical clustering methods focused on the whole genome information instead of previous selected genes to overview of the signature of gene networks in phenotypes which can avoid bias and subject judgement.^12^ Weighted Gene Co‐expression Network Analysis has been widely used in the study of multiple diseases.^13^, ^14^, ^15^ By constructing a co‐expression network of genes and an identification module, WGCNA can investigate hub genes closely related to clinical phenotypes, which will provide us a beacon of hope for discovering new molecular biomarkers and therapeutic targets in GBM.

In the present study, we acquired the clinical information of GBM patients from The Cancer Genome Atlas (TCGA) database. These patients were defined as high‐ and low‐risk group respectively in accordance with the follow‐up time and survival status. The gene co‐expression networks of these two groups were constructed by WGCNA, then the modules related to prognosis were identified and the core genes in the modules were obtained. Through the screening and functional enrichment analysis of the hub genes in the prognosis‐related specific modules, two genes, cell division cycle associated 5 (CDCA5) and cell division cycle associated 8 (CDCA8), which are vital to the prognosis of GBM patients have been selected. Additionally, we performed a single‐gene analysis of CDCA5 and CDCA8 to further validate our prediction. These findings may greatly help us develop new therapeutic targets and improve GBM patient's clinical outcomes.

## 2 MATERIALS AND METHODS

### 2.1 Data acquisition and samples grouping

The Cancer Genome Atlas database (https://www.cancer.gov/about‐nci/organization/ccg/research/structural‐genomics/tcga) is a landmark cancer genomics database, which mainly contains clinical data of various human cancers, such as genome variation, mRNA expression, miRNA expression, methylation and other data. Our study included a total of 142 GBM patients with complete clinical information from TCGA database. Patients were divided into high‐ and low‐risk group according to the follow‐up time and survival status. A total of 93 patients with a follow‐up of less than 60 months and the survival status of death were defined as high‐risk patients, while 49 patients of the rest were defined as low‐risk patients. We downloaded and used TCGA level 3 FPKM RNA‐seq and clinical data for subsequent WGCNA network construction.

### 2.2 WGCNA co‐expression network construction and significant module identification

In our study, we constructed the gene co‐expression network of high‐ and low‐risk GBM patients respectively via the standard procedure of WGCNA.^10^ The WGCNA R package (http://www.r‐project.org/) was used for WGCNA installation, data reading and import. The data were obtained by removing genes with zero variance between groups and including the first 75% of gene sets with Median Absolute Deviation (MAD) for further analysis. The filtering principle of soft threshold was to make the constructed network more consistent with the characteristics of scale‐free network. The weighted adjacency matrix was transformed into a topological overlap matrix (TOM) to estimate its connectivity in the network. The hierarchical clustering method was used to construct the clustering tree structure of the TOM. Different branches of the cluster tree represented different gene modules, and different modules were represented by different colours.

Based on their weighted correlation coefficients, genes were classified on the grounds of their expression patterns. Finally, genes were divided into multiple modules according to gene expression patterns. Comparing the co‐expression networks of the high‐ and low‐risk GBM patients, the module with the minimum value of preservation *Z*‐summary score was the specific module which could distinguish high‐ and low‐risk group. We identified the hub gene of this non‐preserved module by the degree of genes linkage and performed functional enrichment analysis on them.

### 2.3 Functional enrichment analysis of hub genes

The cluster Profiler package in R ^16^ was used to annotate hub genes to fully discover and explore their functional correlations. Gene ontology (GO) was used to assess the relevant functional categories. The *P*‐value of <.05 and *q*‐value of <.1 were set as the threshold.

### 2.4 Validation of the interest genes with external database

We used external databases to verify the interested genes. The relationship between the genes of interest and the prognosis of GBM patients was obtained through the PrognoScan database ^17^ (http://dna00.bio.kyutech.ac.jp/PrognoScan/index.html) from the gene expression data of GSE 4412. Then we used the TCGA database to analyse the difference in expression of genes of interest.

### 2.5 Transfection

All RNA duplexes were synthesized by Vigene Company (China). The corresponding sequences are listed as follows: si‐CDCA5#1:sense, 5′‐GGCCAUGAAUGCCGAGUUUTT‐3′ and antisense, 5′‐AAACUCGGCAUUCAUGGCCTT‐3′; si‐CDCA5#2: sense, 5′‐GCAGUUUGAUCUCCUGGUUTT‐3′ and antisense, 5′‐AACCAGGAGAUCAAACUGCTT‐3′; si‐CDCA5#3: sense, 5′‐CGCAG GAGCCCUAGGAUUUTT‐3′, and antisense, 5′‐AAAUCCUAGGGCUCCUGCGTT‐3′; si‐CDCA8#1:sense, 5′‐GUGGAAAUACGAAUCAAGCTT‐3′, and antisense, 5′‐GCUUGAUUCGUAUUUCCACTT‐3′; si‐CDCA8#2: sense, 5′‐UUGACUCAAGGGUCUUCAATT‐3′, and antisense, 5′‐UUGAAGACCCUUGAGUCAATT‐3′; si‐ CDCA8#3: sense, 5′‐CCAAAACACGAAAGGUAAUAC‐3′, and antisense, 5′‐AUUACCUUUCGUGUUUUGGCA‐3′; A negative control siRNA (si‐NC) was also used: sense, 5′‐UUCUCCGAACGAGUCACGUTT‐3′ and antisense, 5′‐ACGUG ACUCGUUCGGAGAATT‐3′. U87‐MG GBM cells were transfected with si‐NC, si‐CDCA5, and si‐CDCA8 using the Lipofectamine 2000 according to the manufacturer's instructions.

### 2.6 Quantitative real‐time polymerase chain reaction (qRT‐PCR)

The mRNA levels of CDCA5 and CDCA8 in U87‐MG cells were analysed by qRT‐PCR. After transfection, all cells were extracted total RNA by using TRIzol reagent. Then, reverse transcription was performed with a reverse transcription assay kit following the manufacturer's instructions (Applied Biosystems). Amplification was performed using SYBR Green All‐in‐one qPCR Mix (GeneCopoeia). The following thermocycling protocol was used: pre‑denaturation at 95°C for 30 seconds, 40 cycles of 15 seconds at 95°C, 30 seconds at 60°C, and melting was done at 65°C. The primers were: CDCA5 forward, 5′‐ AAATCTGGCCGAAGACACCC‐3′ and reverse, 5′‐ CATGGG CCACGATCCTCTTT‐3′; and CDCA8 forward, 5′‐CCTGACACCCAGGTTTGAC T‐3′ and reverse, 5′‐ GCAATACTGTGCCTCTGCAA‐3′; and GAPDH forward, 5′‐GAGAAGGCTGGGGCTCATTT‐3′ and reverse, 5′‐TAAGCAGTTGGTGGTGCA GG‐3′. Expression data were normalized to the expression of GAPDH with the 2^−ΔΔCt^ method.

### 2.7 Cell counting kit (CCK)‐8 assay

Cells were seeded in 96‐well plates. CCK‐8 assay was performed at 24, 48, 72 and 96 hours according to the manufacturer's instructions. Absorbance was detected at 450 nm using microplate reader.

### 2.8 Colony formation assay

Cells were seeded into six‐well plates (500 cells/well) and incubated in humidified air containing 5% CO_2_ at 37°C for 12 days. Culture medium was replaced every 2‐3 days. The former colonies were washed with PBS, fixed with 4% paraformaldehyde and then stained with 0.01% crystal violet. The numbers of colonies were counted.

### 2.9 Apoptosis analysis

Cells were seeded in a 6‐well plate (2 × 10^5^ cells/well). Cells were harvested by trypsin and washed twice with PBS. Apoptosis was analysed using Annexin V‐FITC Apoptosis Detection Kit according to the manufacturer's instructions. The apoptosis cells were measured by staining with Annexin V‐FITC along with Propidium Iodide. After incubating for 15 minutes, the stained cells were detected.

### 2.10 Further single‐gene analysis of the interest genes in hub genes

This study intended to further tap the relationship between interest genes in hub genes and GBM progression through single‐gene analysis. Firstly, we analysed all RNA‐seq data on GBM in the TCGA database. The correlation coefficient of >.4 and the *P*‐value of <.001 were set as the filtering condition. Then we obtained the genes co‐expressed with the interested genes. After screening the genes related to the p53 signalling pathway, the ‘pheatmap’ and ‘gplot’ packages were used to draw the heatmap for the correlation analysis of the interested genes. At last, the relationship with immune microenvironment was obtained by TIMER ^18^ (Tumour Immune Estimation Resource, https://cistrome.shinyapps.io/timer/). All statistical analyses were conducted using R 3.6.0 (https://www.r‐project.org/).

## 3 RESULTS

### 3.1 Data pre‐processing and soft threshold screening

**FIGURE 1:**
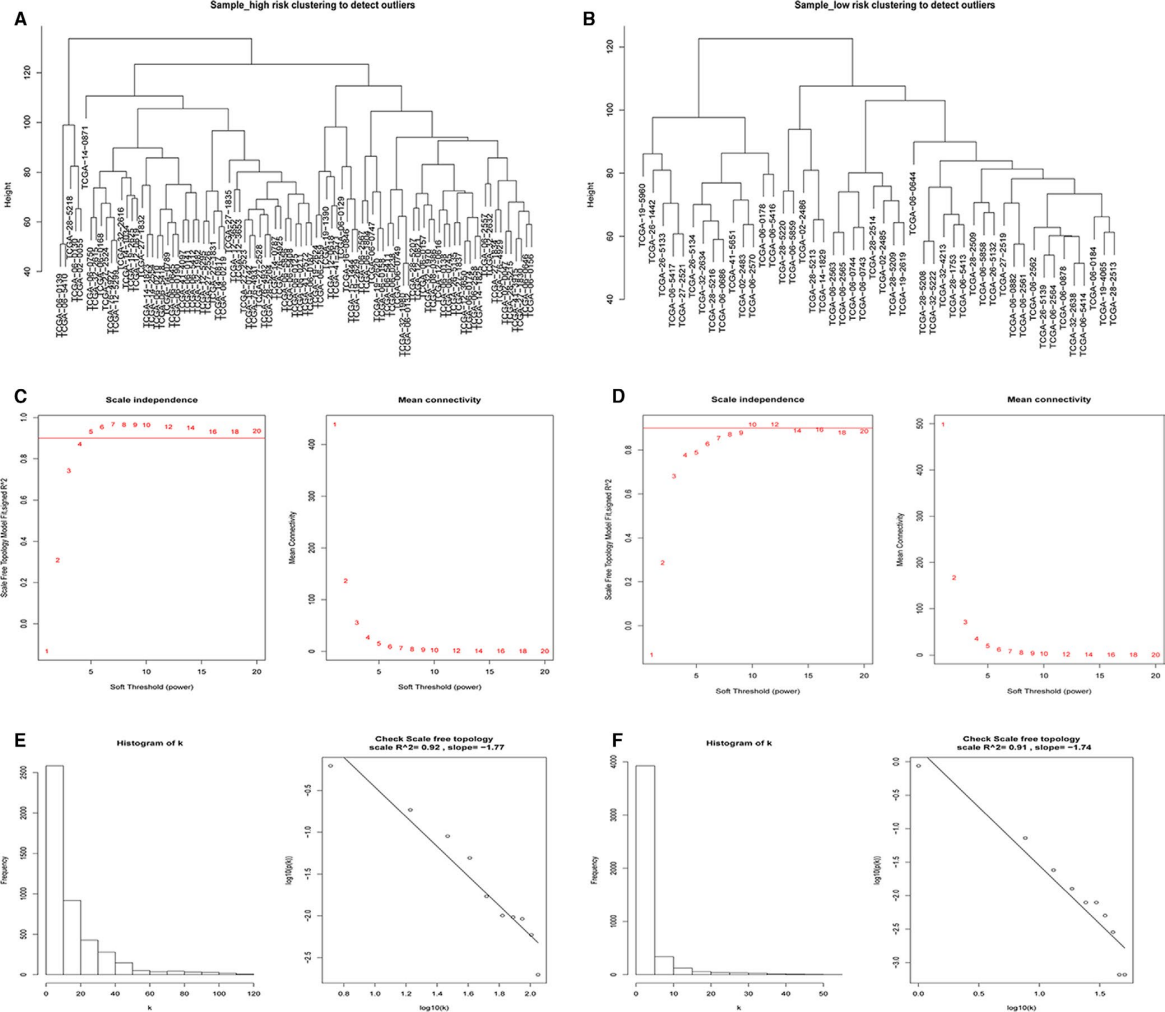
The detection of outlier samples, the selection and validation of optimal soft threshold power to construct gene co‐expression networks. A, The clustering dendrogram of high‐risk samples to detect outliers. B, The clustering dendrogram of low‐risk samples to detect outliers. C, The scale independence and the mean connectivity of the WGCNA analysis of the high‐risk samples. D, The scale independence and the mean connectivity of the WGCNA analysis of the low‐risk samples. E, The histogram of k and the correlation coefficient between k and p (k) of the high‐risk samples. F, The histogram of k and the correlation coefficient between k and p (k) of the low‐risk samples

Considering the sensitivity of WGCNA to the effect of batch processing. We first pre‐processed the data sets of all 93 GBM patients in the high‐risk group and 49 GBM patients in the low‐risk group from the TCGA database. By removing genes with zero variance between groups and including the first 75% of gene sets with MAD, we obtained the gene sets for following analysis. Subsequently, we used the hclust function to confirm the effect of batch removal from the dataset and to see if there were any outliers. The results showed that these datasets had not been corrected due to the batch removal effect, and the tree graph and samples were clustered in the correct random order (Figure 1A,B). Due to the premise of WGCNA algorithm needs to assume that gene network is subject to scale‐free distribution. Thus, we next needed to screen out appropriate soft threshold (power) to make the constructed network more consistent with the characteristics of scale‐free network. We set the soft threshold as 5 (high‐risk group) and 10 (low‐risk group), respectively to meet the selected criteria of power value (Figure 1C,D). By calculating the scale‐free topology fitting index, the value of R‐square reached 0.9 (Figure 1E,F). This result further verified and illustrated the feasibility of WGCNA.

### 3.2 Construction of co‐expression networks and identification of modules

**FIGURE 2:**
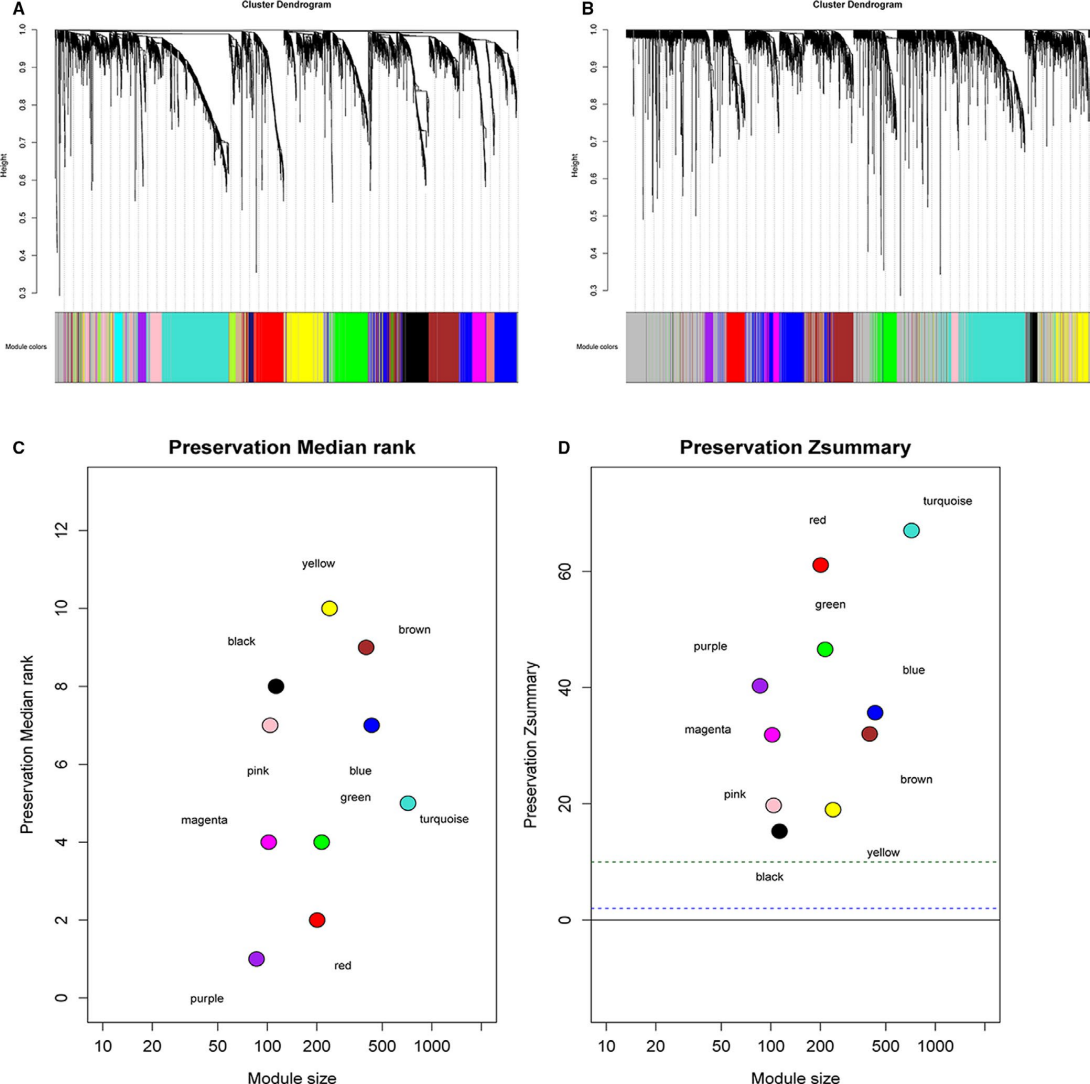
Co‐expression modules identified and characterized by WGCNA. Clustering dendrograms of (A) High‐risk samples and (B) Low‐risk samples. (C) The preservation median rank of ten co‐expression modules. (D) The preservation *Z*‐summary score of ten co‐expression modules

We constructed two co‐expression networks of the high‐ and low‐risk GBM patients. Hierarchical clustering analysis was conducted based on weighted correlation, and the clustering results were segmented according to the set criteria to obtain different gene modules (Figure 2A,B). The results showed that the module tags were still clustered together in the high‐risk group network, indicating that the preservation of this module was well. By using WGCNA for the low‐risk group, we identified ten modules of different sizes, and used branches of the cluster tree and different colours to represent them. Then the high‐risk group network was mapped to the low‐risk group network modules. This approach helped us identify non‐preserved modules. Non‐preserved modules could explain the change of network properties between low‐ and high‐risk group networks. In addition, these non‐preserved modules may be related to survival status of GBM patients and tumour progression. To validate the stability of WGCNA, we used the module preservation function to calculate the module preservation. The saved median and *Z*‐summary score were showed for different colour modules (Figure 2C,D). The turquoise module had the highest *Z*‐summary score, which indicated that it retained the network characteristics of the high‐risk group network. However, the black module with the lowest *Z*‐summary score meant a low degree of preservation, indicating that the prognosis level could be distinguished between high‐ and low‐risk patients.

### 3.3 Identification and functional enrichment analysis of hub genes

**FIGURE 3:**
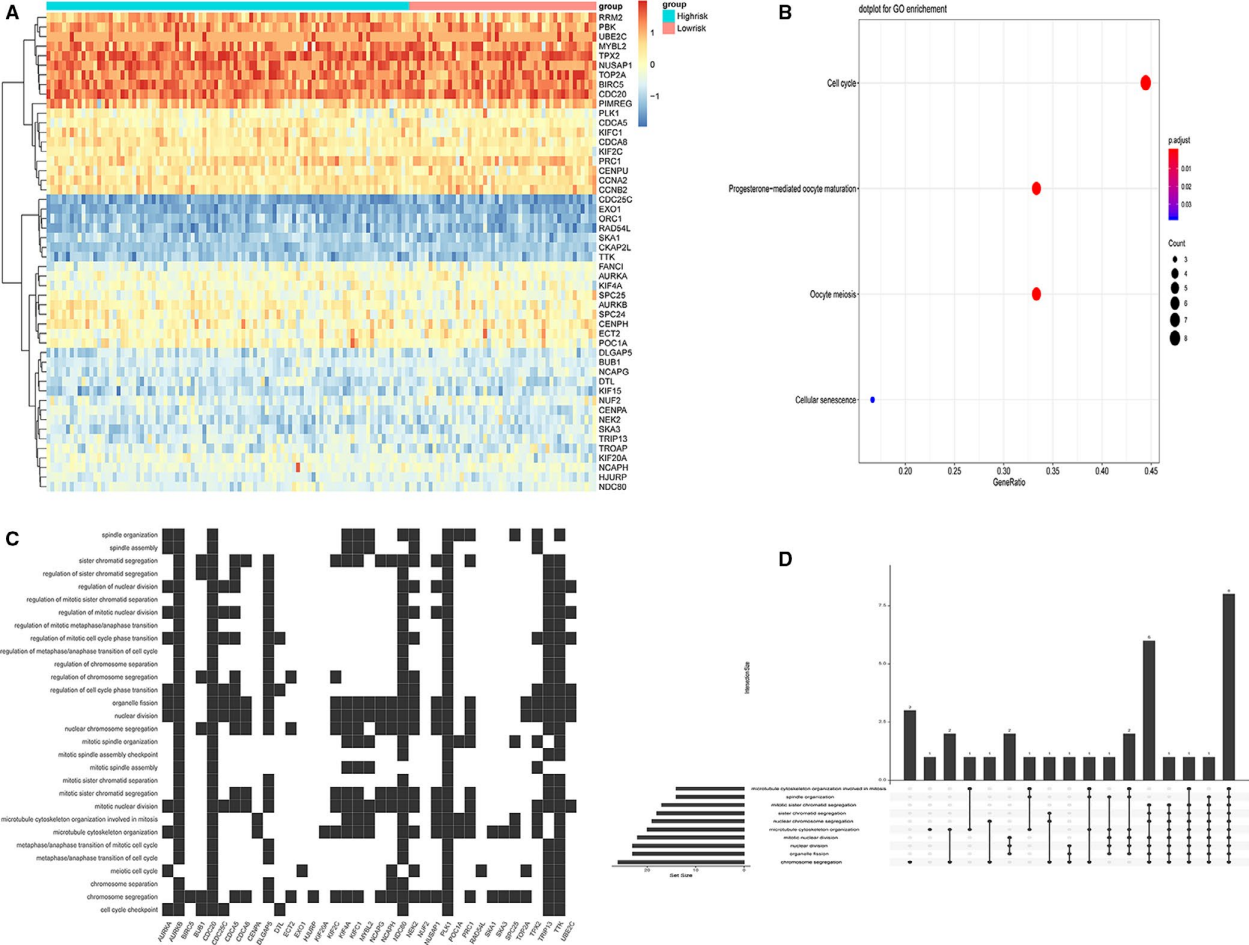
Drawing of the hub genes heat map and performing GO enrichment analysis in the black module. A, The heat map of 50 hub genes in the black module. B, The bubble plot showed the top enriched biological process of these genes. C, The scatter plot showed the distribution of hub genes involved in specific links in the cell cycle. D, The Up‐Set plot showed the interactions among the ten links of cell cycle

To identify key nodes associated with prognosis, we performed a more detailed analysis of the black module. Because it was minimally preserved between networks and could be used to distinguish between samples of high‐ and low‐risk GBM patients. As a result, a heat map of 50 core genes was obtained (Figure 3A), which may play an important role in shortening the survival of GBM patients. Then the Cytoscape software was used to calculate the strength of the intramodule connectivity of each gene for the non‐preserved modules. We sorted by score and finally obtained the top 10 hub genes (CDC20, NCAPH, CDCA5, BUB1, CDCA8, PBK, KIF2C, TPX2, TTK and TOP2A).

Gene ontology enrichment analysis was performed on the genes in the black module. The results demonstrated that the biological process of these 50 core genes mainly enriched in three aspects: Cell cycle, Progesterone‐mediated oocyte maturation and Oocyte meiosis (Figure 3B). In addition, we showed in detail that each gene corresponded to a specific link in the cell cycle (Figure 3C). By further enrichment analysis of genes and the interactions among the ten links in cell cycle, we found that these genes mainly played important roles in processes of chromosome segregation, organelle fission, nuclear division and mitotic nuclear division (Figure 3D).

### 3.4 Validation of the interest genes with external databases

**FIGURE 4:**
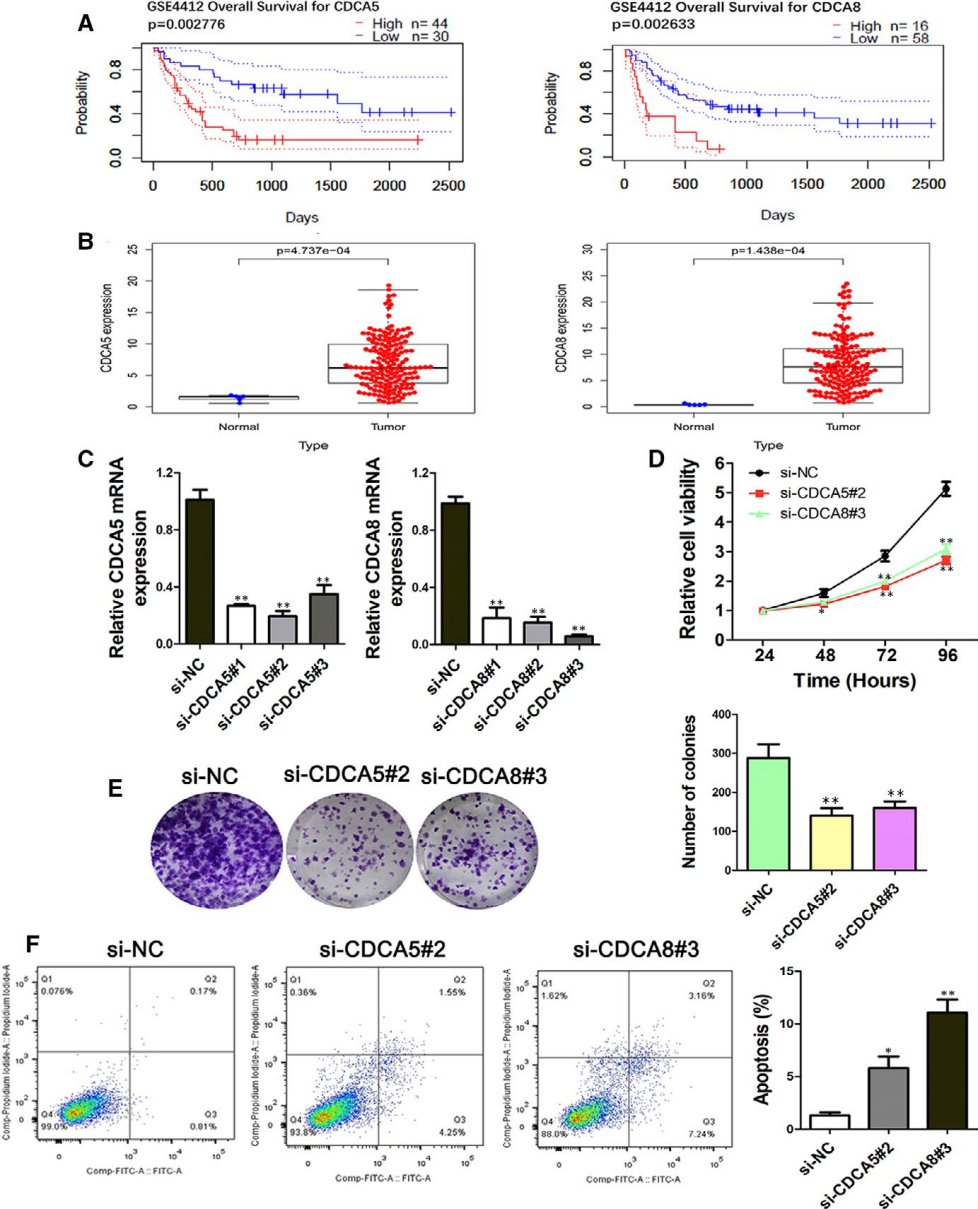
Validation with external databases and functional analysis of CDCA5 and CDCA8 in GBM cells. A, The overall survival rate of high‐ and low‐risk patients based on the expression levels of CDCA5 and CDCA8 in the GSE4412 dataset. B, The difference in expression of CDCA5 and CDCA8 between normal and tumour group based on the TCGA database. C, qRT‐PCR experiments were performed to detect the expression of CDCA5 and CDCA8 after siRNA transfection. D, Cell proliferation ability (E) clonogenicity and (F) apoptosis of U87‐MG cells after transfection by si‐CDCA5 and si‐CDCA8 was determined. *P < .05, **P < .01 vs si‐NC

Through searching literature for the top 10 screened core genes, we found that there were few reports about the mechanism of CDCA5 and CDCA8 with GBM. Thus, they were expected to be new biological targets for the treatment of GBM. Firstly, gene expression profiles of CDCA5 and CDCA8 were obtained from the NCBI GEO database (https://www.ncbi.nlm.nih.gov/geo/): GSE4412. GBM Patients were divided into high and low expression groups according to the CDCA5 and CDCA8 gene expression levels. The PrognoScan database was used to analyse their relationship with the prognosis of GBM patients. The results showed a significant difference in overall survival (OS) between the two groups, the OS in both CDCA5 and CDCA8 high expression groups were dramatically shortened when compared with the low expression groups (Figure 4A). Secondly, we used the TCGA database to analyse the difference in expression of CDCA5 and CDCA8. Both were significantly overexpressed in the tumour group compared to the normal group (Figure 4B).

### 3.5 Effect of CDCA5 and CDCA8 on proliferation, clonogenicity and apoptosis of GBM cells

To further study the function of CDCA5 and CDCA8 in GBM, corresponding siRNAs which targeted the CDCA5 and CDCA8 genes were specifically designed. qRT‐PCR analysis showed that the relative mRNA expression of CDCA5 and CDCA8 in U87‐MG cells in the si‐gene group was lower than the si‐NC group (Figure 4C). The most effective siRNAs (si‐CDCA5#2 and si‐CDCA8#3) were used for all subsequent experiments. After knockdown of CDCA5 and CDCA8 by siRNA, the cell viability of U87‐MG cells was significantly decreased when compared with the NC group (Figure 4D). To assess the long‐term effects of CDCA5 and CDCA8 on U87‐MG cells proliferation, the colony formation assay was performed. Figure 4E revealed that CDCA5 and CDCA8 genes knockdown markedly diminished the number of colonies. Further, we evaluated the effect of CDCA5 and CDCA8 on the apoptosis of U87‐MG cells. The apoptosis of cells was detected by flow cytometry. As shown in Figure 4F, the apoptosis rates of cells in si‐CDCA5 and si‐CDCA8 groups were significantly higher than those in si‐NC group. The above results indicating that inhibiting CDCA5 and CDCA8 expression could inhibit proliferation, clonogenicity and promote the apoptosis of GBM cells.

### 3.6 Single‐gene analysis of CDCA5 and CDCA8

**FIGURE 5:**
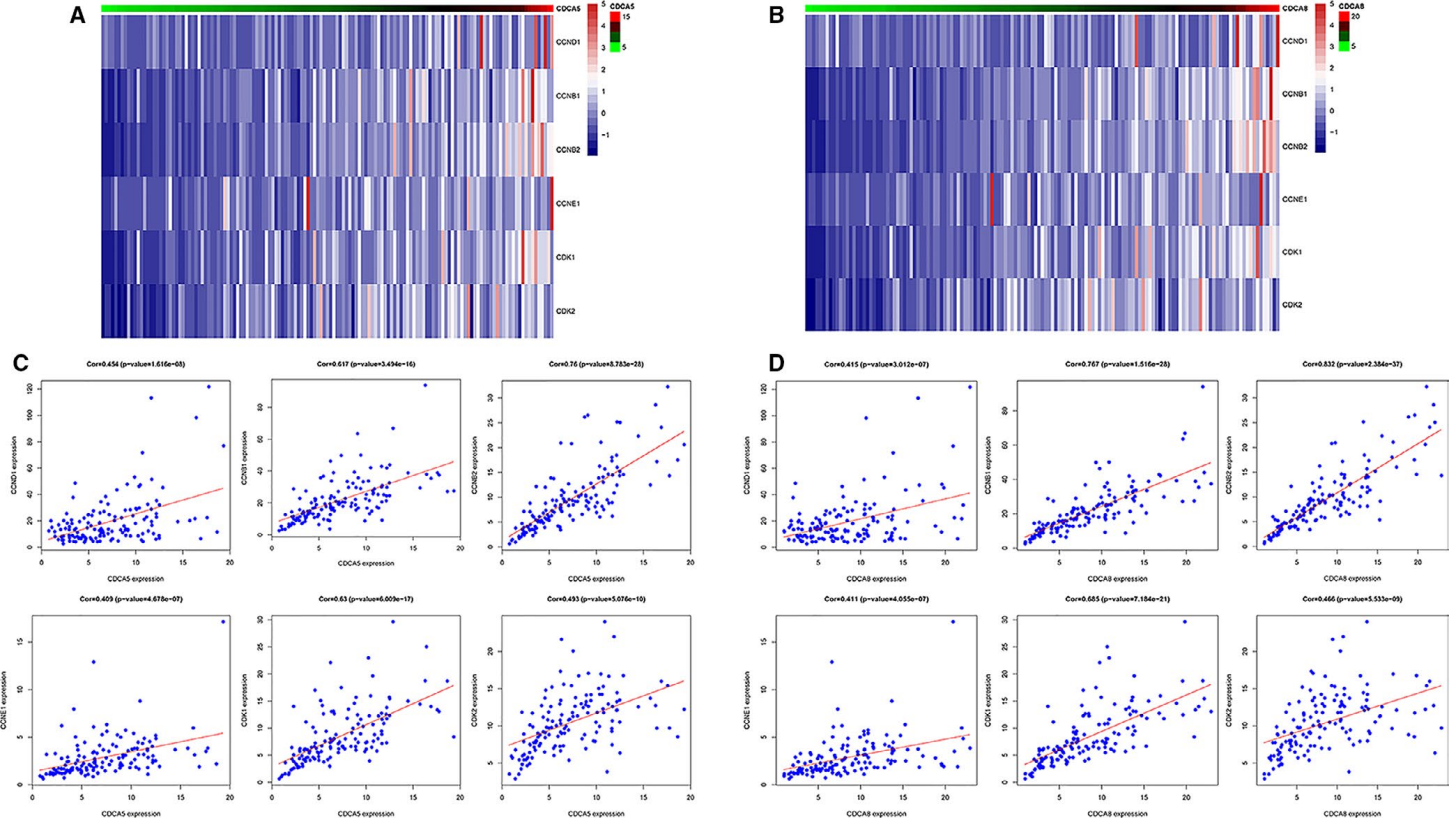
Single‐gene analysis to study the relationship between CDCA5 and CDCA8 and cell cycle related genes in the p53 pathway. A and B, The heatmaps of CDCA5 and CDCA8 and cell cycle related genes expression profiles in the p53 pathway. C, The scatter plot showed the correlation between CDCA5 and cell cycle related genes in the p53 pathway. D, The scatter plot showed the correlation between CDCA8 and cell cycle related genes in the p53 pathway

**FIGURE 6:**
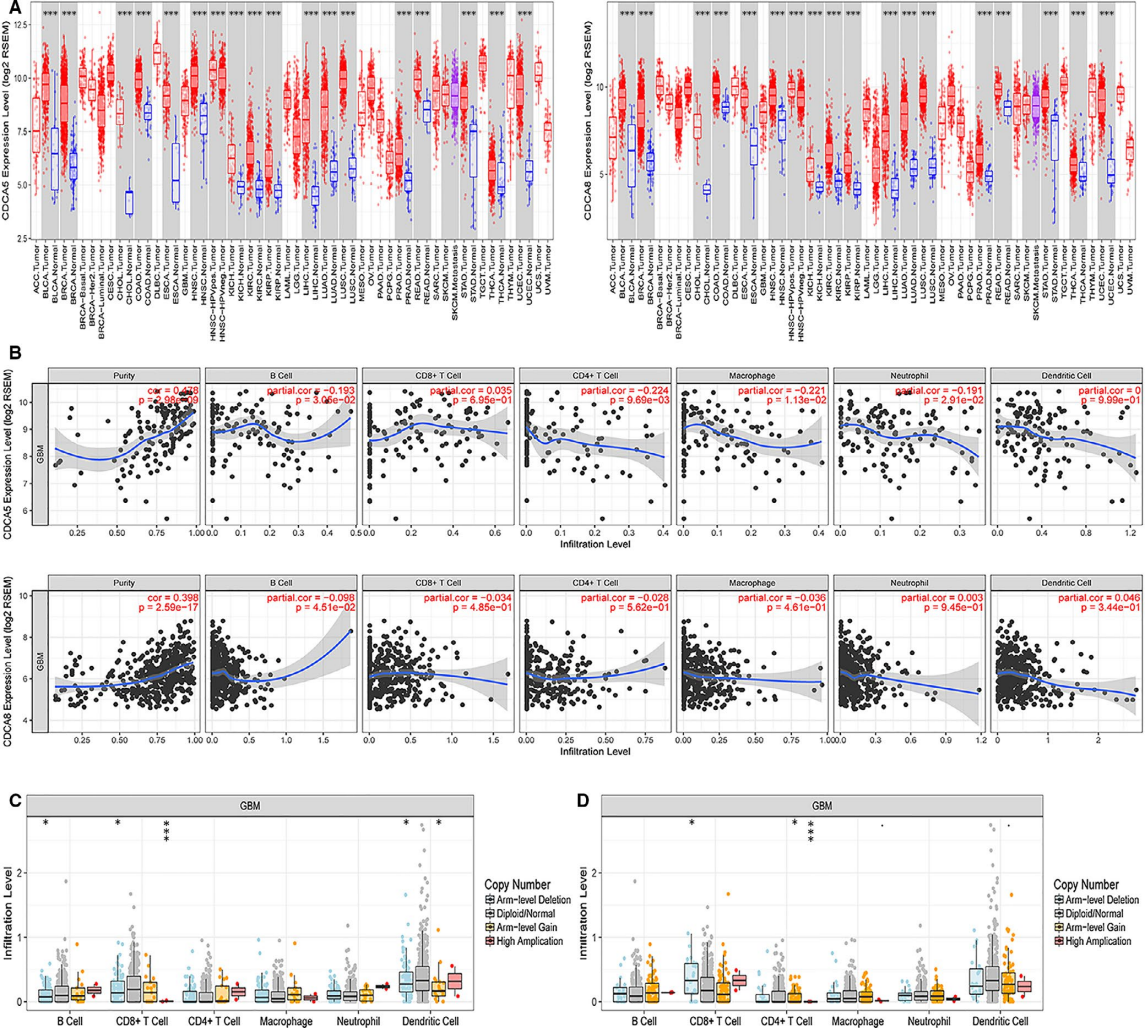
Single‐gene analysis to study the relationship between CDCA5 and CDCA8 and immune microenvironment. A, The differential expression of CDCA5 and CDCA8 in tumours and normal tissues of multiple cancer species. B, The relationship between the expression levels of CDCA5 and CDCA8 and the six types of immune cells. The relationship between expression levels and copy number variations of (C) CDCA5 and (D) CDCA8 and the levels of infiltration of six immune cells

We further performed single‐gene analysis on CDCA5 and CDCA8 at two aspects to explore their role in the occurrence and development of GBM. The co‐expression analysis of CDCA5 and CDCA8 with the whole genome of TCGA database were conducted to screen the gene correlations related to cell cycle in the p53 signalling pathway. The correlation analysis heatmaps were made by the "pheatmap" and" gplot "packages (Figure 5A,B). We found that both of these two genes had good correlations with genes that regulate the cell cycle in the p53 signalling pathway: CCND1, CCNB1, CCNB2, CCNE1, CDK1, and CDK2 (Figure 5C:CDCA5 and Figure 5D:CDCA8). These results suggested that CDCA5 and CDCA8 may be involved in the signal regulation of p53 pathway by affecting relevant genes in the cell cycle. Then, the relationship between these two genes and the immune microenvironment of GBM was obtained by TIMER. We studied the differential expression of the CDCA5 and CDCA8 in tumours and normal tissues of multiple cancer species, the relationship between expression levels and copy number variations of genes and the levels of infiltration of six immune cells (B cell, CD8^+^ T cell, CD4^+^ T cell, macrophage, neutrophil, dendritic cell). The results revealed that CDCA5 and CDCA8 were differentially expressed in tumour and normal tissues of multiple cancer species, showing a tendency of up‐regulation (Figure 6A). Figure 6B showed that there was no significant correlation between the expression levels of CDCA5 and CDCA8 and the six types of immune cells. However, the high amplification of CDCA5 was obviously correlated with CD8^+^ T cells in GBM (Figure 6C). Similarly, the high amplification of CDCA8 had a significant correlation with CD4^+^ T cells in GBM (Figure 6D). It was indicated that CDCA5 and CDCA8 may affect the immune microenvironment of GBM through this mechanism, leading to the malignant progression of GBM.

## 4 DISCUSSION

Glioblastoma is the most common primary malignant brain tumour in adults, with a poor prognosis and high mortality due to its highly aggressive characteristics.^19^, ^20^, ^21^ The advances in GBM therapy have not concomitant with prominent amelioration in outcomes until recently.^22^ Therefore, exploring molecular targets and therapeutic means are urged needed.^23^ Years of molecular studies have identified many key links that affect the development and progression of GBM.^24^ Especially with the progress of high‐throughput genome technology make it possible to find more potential molecular markers by using bioinformatics methods.

In the present study, data of GBM patients with complete clinical information were obtained from TCGA database. The patients were divided into high‐risk and low‐risk groups according to the follow‐up time and survival status of the patients. Our study was the first to construct co‐expression modules related to survival by WGCNA in the two groups of patients. Compared with other methods, WGCNA has many obvious advantages. Because its analysis focuses on the association between co‐expression modules and clinical features of interest, the analytical results have better reliability and biological significance.^25^ We analysed the preservation of all modules in the high‐risk group and low‐risk group. Due to the low degree of preservation between the high‐ and low‐risk group, the non‐ preserved module of the two co‐expression networks (that was, the module with the minimum preservation *Z*‐summary score) was able to distinguish the prognosis level between the two groups.

The preservation *Z*‐summary score results from Figure 2D showed that the black module was identified to be the lowest conservative module due to its lowest *Z*‐summary value. Therefore, we focused on the black module to explore the influencing factors related to survival of patients.

We identified 50 key genes from the black module and plotted a heat map. These genes were the key genes that affect the survival time and survival status of patients with GBM. Our further analysis of these genes by GO showed that these prognostic genes were mainly related to cell cycle. In particular, it was most closely related to the key links: chromosome segregation, organelle fission and nuclear division. Therefore, we speculated that these genes may influence the cell cycle and accelerate the replication of cancer cells by regulating the cell cycle of cancer cells, resulting in the rapid spread of cancer cells. In order to explore the specific mechanism of the effect of these genes on survival, we screened the top 10 genes (CDC20, NCAPH, CDCA5, BUB1, CDCA8, PBK, KIF2C, TPX2, TTK and TOP2A). By retrieving related literature, we found that CDCA5 and CDCA8, as important regulatory proteins in the cell cycle in cancer, were recognized as oncogenes.^26^, ^27^, ^28^, ^29^ However, compared with other genes, there scarcely no reports about the mechanism of CDCA5 and CDCA8 with GBM. Thus, conduct study on the specific mechanism of CDCA5 and CDCA8 to GBM malignant progression might have vital clinical significance.

After verification of the two selected genes through external databases, we found that: the OS of patients with high expression of CDCA5 and CDCA8 in tumour tissues were significantly decreased from the chip data of GSE4412. Moreover, the difference analysis resulted from the TCGA database discovered that CDCA5 and CDCA8 were significantly highly expressed in GBM patients. These results were consistent with the two genes on the survival time of patients in other types of tumours.^30^, ^31^, ^32^ Further experimental data demonstrated that silencing the CDCA5 and CDCA8 would influence the biological behaviours of GBM cells. Then, we carried out single‐gene analysis of CDCA5 and CDCA8, respectively to further explore their potential mechanism in the development of GBM. We tried to explain the effects from two aspects: molecular mechanism and tumour immunity. We analysed the correlation between the two and all genes involved in the p53 signalling pathway. p53, as a star tumour suppressor gene, can regulate cell cycle and prevent cell cancerization. It is referred to as the ‘guardian of the genome’ by the scientific community. Generally, more than 50% of cancer patients have mutations and inactivation of p53 gene.^33^, ^34^ The p53 signalling pathway, as the most influential signalling pathway in the tumour field, has a significant impact on the incidence of different cancers.^35^ In particular, it plays an important role in regulating cell cycle. We were pleased to find that CDCA5 and CDCA8 were significantly correlated with CCND1, CCNB1, CCNB2, CCNE1, CDK1 and CDK2 (correlation coefficient >.4, *P* < .05). These proteins were key proteins that regulating the cell cycle in the p53 signalling pathway. CCND1 is a protein encoded by the human CCND1 gene. It forms a complex with CDK4 or CDK6 and acts as a regulatory subunit, which is essential for the transition from G1 to S phase of cells. Mutations, amplification or overexpression of the gene could change the cell cycle process. These phenomena often occurred in many tumours and may cause tumorigenesis.^36^, ^37^ CCNB1/2, as a vital member of the cyclin family, is an important cell cycle regulator related to G2/M detection points in cells. It regulates cyclin‐dependent kinase 1 (CDK1) and forms a complex with it to phosphorylate the substrate, initiate cells from G1/S phase to G2/M phase, and promote mitosis. Plenty of evidence indicates that CCNB1/2 dysfunction is an early event in tumorigenesis, and its unregulated expression could be observed in many human tumours, including breast cancer, lung cancer and brain cancer.^38^, ^39^ CCNE1 plays an important role in regulating the cell from G1 to S phase. It forms a complex by binding and activating CDK2, which plays a very important role in inducing the synchronization of DNA replication, centrosome replication and regulation, chromosome reconstruction and histone synthesis. It has been reported in the literature that the high expression of CCNE1 was closely related to the poor clinical prognosis of patients with various malignancies such as ovarian, bladder and colon cancer.^40^, ^41^ Therefore, we speculated that the effect of CDCA5 and CDCA8 on the prognosis of GBM patients may be achieved by participating in the regulation of cell cycle in the p53 pathway. In particular, the co‐expression of these key genes leaded to dysfunction at G1/S checkpoint and/or G2/M checkpoint through overexpression of these genes, leading to active replication of cancer cells and malignant tumour progression.

In recent years, tumour immunotherapy has become a novel focus in cancers. More and more studies have focused on the infiltration of immune cells in tumour tissues to explore the relationship between tumour microenvironment and clinical outcomes.^42^, ^43^ By applying the online tool of TIMER, we attempted to explore the relationship between CDCA5 and CDCA8 and immune cells in GBM. This present study revealed that CDCA5 and CDCA8 with high amplification had significant effects on CD8^+^ T cell and CD4^+^ T cell, respectively in GBM patients under different gene copy states. Thus, both of these two genes may also influence the prognosis to some extent by regulating the immune microenvironment of GBM patients. However, the specific relationship between these two genes and immunity of GBM need to be further explored.

## 5 CONCLUSION

Our study used WGCNA to construct co‐expression modules related to the survival of GBM patients. We identified the non‐preserved module and hub genes associated with poor prognosis in GBM patients. CDCA5 and CDCA8 were screened out as the genes of interest and verified its roles in the GBM cells. We found the role of the CDCA5 and CDCA8 in regulating the cell cycle in the p53 pathway, and explain their potential pathways and molecular mechanisms. In addition, this study revealed the effects of CDCA5 and CDCA8 in the immune microenvironment of GBM. It provided new molecular targets and intervention strategy for improving the prognosis of GBM patients.

## CONFLICT OF INTEREST

The authors declare no conflict of interest.

## AUTHOR CONTRIBUTIONS

**Jing Zhou:** Data curation (equal). **Hao Guo:** Data curation (equal). **Likun Liu:** Data curation (equal). **Shulan Hao:** Methodology (equal). **Zhi Guo:** Methodology (equal). **Fupeng Zhang:** Methodology (equal). **Yu Gao:** Methodology (equal). **Zhi Wang:** Methodology (equal). **Weiwei Zhang:** Methodology (equal).
